# German claims data analysis to assess impact of different intraocular lenses on posterior capsule opacification and related healthcare costs

**DOI:** 10.1007/s10389-017-0851-y

**Published:** 2017-10-24

**Authors:** Nils Kossack, Christian Schindler, Ines Weinhold, Lennart Hickstein, Moritz Lehne, Jochen Walker, Aljoscha S. Neubauer, Dennis Häckl

**Affiliations:** 1 WIG2 Institute for Health Economics and Health System Research, Leipzig, Germany; 2 InGef Institut für angewandte Gesundheitsforschung, Berlin, Germany; 3 Elsevier Health Analytics, Berlin, Germany; 4 Praxis für Augenheilkunde und Institut für Gesundheitsökonomik, Munich, Germany

**Keywords:** Cataract, Acrylic hydrophobic/hydrophilic intraocular lens, Posterior capsule opacification, Follow-up costs, Neodymium-doped yttrium-aluminum-garnet (Nd:YAG) laser capsulotomy, I10, I13

## Abstract

**Aim:**

Cataract extraction is one of the most frequent surgeries in Germany. In most cases, the clouded natural lens is replaced by a hydrophobic or hydrophilic acrylic intraocular lens (IOL) implant. The most common long-term complication after cataract surgery is the development of a posterior capsule opacification (PCO). Although no precise real world data are available, published evidence suggests a lower risk for PCO development for hydrophobic acrylic IOLs compared to hydrophilic acrylic IOLs. Therefore, in the present study we assessed real world data on the impact of different IOL material types on the incidence of post-operative PCO treatment.

**Subject and methods:**

In this retrospective study, we included 3,025 patients who underwent cataract extraction and implantation of either an acrylic hydrophobic or hydrophilic IOL in 2010. We assessed clinical outcomes and direct costs in a 4-year follow-up period after cataract surgery from a statutory health insurance (SHI) perspective in Germany.

**Results:**

PCO that required capsulotomies occurred significantly (*p* < 0.0001) less frequent in patients who had received a hydrophobic IOL (31.57% of 2,078 patients) compared to the group with hydrophilic IOL implants (56.6% of 947 patients) and costs per patient for postoperative treatment in a 4-year follow-up were 50.03 € vs. 87.81 € (i.e. 75% higher in the latter group, *p* < 0.0001).

**Conclusion:**

Considering the high prevalence of cataract, the economic burden associated with adverse effects of cataract extraction is of great relevance for the German SHI. Hydrophobic lenses seem to be superior regarding both medical and economic results.

## Introduction

Cataract, the clouding or loss of transparency of the eye’s natural lens is the leading cause for blindness worldwide (Abraham et al. 2006). Prevalence rates of age-related cataract as the most common form considerably increases from about 30% for the 60–69-year-old population up to more than 60% for those older than 70 years (Prokofyeva et al. 2013). Up to now, surgical cataract extraction is the sole effective treatment to restore visual function and prevent blindness (Prokofyeva et al. 2013). With about 850,000 up to 1,000,000 yearly cases, cataract extraction is one of the most frequently performed surgeries in Germany, representing a high economic burden to the healthcare system (AQUA - Institut für angewandte Qualitätsförderung und Forschung im Gesundheitswesen GmbH 2010). During the procedure, the affected lens is extracted and replaced by an IOL implant. IOLs are available in four optic materials, i.e. polymethylmethacrylate (PMMA), high water content hydrophilic acrylic, low water content hydrophobic acrylic and hydrophobic silicone. Up to now, hydrophobic acrylic is the most frequently implemented IOL material (Lundström et al. 2012).

### Clinical and economic impact of complications after cataract surgery

Risks of cataract surgery itself (e.g. posterior capsular rupture, zonular dehiscence or suprachoroidal hemorrhage) are generally low (AQUA - Institut für angewandte Qualitätsförderung und Forschung im Gesundheitswesen GmbH 2010; Chan et al. 2010; Lundström et al. 2012). Postsurgical complications such as changes in intraocular pressure, inflammations (i.e. uveitis, endophthalmitis) or a corneal edema occur sometimes (<0.4%) within a short time frame after surgery (Lundström et al. 2012). Also there is a risk of 0.9% for a retinal detachment in the first 4 years (Chan et al. 2010); therefore, a treatment of glaucoma, a vitreoretinal intervention or an explantation with secondary implantation of an IOL could rarely occur. The by-farmost frequent long-term complication after cataract surgery is a PCO, which usually occurs a few weeks up to several years after cataract surgery (Findl et al. 2007). PCO results in a decreased visual acuity, impairing the patient. In addition, PCO causes impaired contrast sensitivity and glare disability (Nibourg et al. 2015). A development of PCO within a maximum of 1 year after cataract extraction is estimated at 4.2% (Greenberg et al. 2011). Within a 2–4 years’ time frame, an incidence rate of 22.8% (Auffarth et al. 2004) to 38.5% (Fong et al. 2014) has been reported in cataract patients. This so-called secondary cataract can be treated with neodymium-doped yttrium-aluminum-garnet (Nd:YAG) laser capsulotomy, which is generally safe and the standard treatment procedure up to now (Karahan et al. 2014a); however, the Nd:YAG laser treatment of PCO itself can cause secondary effects. Transient increase of an intraocular pressure is observed in 5% of all cases and a decrease in visual acuity in about 4% (Boureau et al. 2009a). Other effects (glaucoma, cystoid macular edema or detachment of the retina) appear in less than 1.5% of laser-treated patients (Karahan et al. 2014b). The main complication is the formation of Elschnig pearls, which affects about 47.60% of the treated patients (Boureau et al. 2009a). On average, a secondary capsulotomy is necessary for every fourth patient (Kato et al. 1997).

Postsurgical complications cause a considerably part of the total costs of cataract treatment (Smith et al. 2005; Boureau et al. 2009a). In the US health system Nd:YAG laser capsulotomy accounts for $500,000,000 annually and is ranked second place in cost statistics for health interventions following cataract surgery itself (Menapace 2007). Billotte and Berdeaux (2004) estimated that in the long-term,^1^ up to 11,500 adverse events based on 400,000 cataract extractions could be avoided if Nd:YAG laser capsulotomy rates could be reduced. To develop effective strategies for PCO prevention, several influencing factors such as material and design of the implanted lens, surgical technique (Pandey et al. 2004), as well as pharmacological management during and post operation are discussed in the literature (Dewey 2006; Wormstone et al. 2009; Chandler et al. 2015; Nibourg et al. 2015). Comparing the available types of IOL materials, PCO development seems to be less likely after hydrophobic acrylic lense implantation compared to hydrophilic acrylic or silicone lens implants (Auffarth et al. 2004; Vasavada et al. 2011; Li et al. 2013; Sundelin et al. 2014). A further factor for the development of PCO seems to be the design of the lens, in favor of sharp-edged compared to round-edged IOLs (Findl et al. 2007; Mencucci et al. 2015).

The present analysis builds on the results of a preliminary study (Kossack et al. 2016). It assesses the impact of two different IOL material types (hydrophobic and hydrophilic acrylate) on the development of PCO rates and other complications related to cataract surgery combined with the associated costs in a 4-year follow-up based on a random sampling from SHI claims data. We performed a retrospective analysis to compare:

1. The incidence of PCO and Nd:YAG laser capsulotomy after cataract extraction in relation to hydrophobic and hydrophilic acrylic IOL implantation in current German practice
2. The associated costs of Nd:YAG laser capsulotomy due to PCO after cataract extraction from a SHI perspective

## Study design/material and methods

### Data and study population

Anonymized claims data were provided by the Institut für angewandte Gesundheitsforschung (InGef). The InGef research database covers approximately 6.7 million insured persons from different German SHIs, mainly company health insurance funds. The external validity of this database compared to German population data has been shown previously (Andersohn and Walker 2016). For the present study, a sample of approximately 4 million insured persons served as study population. This sample is representative for the German population with regard to age and sex for the year 2013. The database includes demographic information, diagnoses, utilization of ambulatory services, hospitalizations and reimbursed drugs as well as remedies and aids on a patient individual level. In Germany, SHI reimbursement of ambulatory services is regulated by the German National Ambulatory Evaluation Scheme (EBM). Utilization of outpatient services can thus be identified by the invoiced fee schedule position numbers (GOP). For historic reasons only, in the region of Bavaria these position numbers differ for the type of implanted IOL, enabling us to differentiate between an implantation of hydrophobic (GOP: 96104A) and hydrophilic (GOP: 96104B) acrylic IOLs. All patient-level data in the InGef database is de-identified to comply with German data protection regulations and German Federal Law; hence, approval of an institutional review board or ethics committee was not required.

The two groups under comparison were selected by a stepwise approach. First, we identified individuals who had been living in Bavaria without interruption and were continuously SHI insured within our study period 2009–2014. Relevant patients were identified with a diagnosis code of *cataract* according to the International Classification of Diseases, Tenth Revision (ICD-10) codes (cataracta senilis: H25, other cataract: H26, diabetic cataract/cataract in other endocrine, nutritional and metabolic diseases/cataract in other diseases classified elsewhere: H28, without other disorders of lens in diseases classified elsewhere: H28.8) and a surgical cataract extraction with an acrylic IOL implant in 2010 (index period). Patients who received both types of acrylic implants as well as patients who already have had an IOL implant before 2010 were excluded from the analysis. In a second step, we divided this sample into two subsamples depending on whether a hydrophobic or hydrophilic acrylic IOL had been implanted after cataract extraction.

### Group comparison

By comparing the two study groups, we analyzed the impact of the implanted IOL material (hydrophobic acrylic versus hydrophilic acrylic) on the development of PCO rates in a 4-year follow-up. The incidence of PCO was identified in patients who underwent capsulotomy, which was identified by documented ICPM (International Classification of Procedures in Medicine) codes for the procedures of *laser capsule polishing* (5–142.0), *surgical capsulotomy* (5–142.1), * Nd:YAG laser capsulotomy* (5–142.2) or *surgical posterior capsule polishing* (5–142.3). To eliminate potential confounders, we additionally considered the following postoperative adverse effects as control variables: changes of intraocular pressure by *treatment of glaucoma* (procedure codes: 5–131, 5–132, 5–133, 5–134), a retinal detachment by *vitreoretinal intervention* (procedure codes: 5–158, 5–159) and an *explantation of the newly implanted IOL with implantation of a next IOL—explantation with secondary implantation of an IOL* (procedure codes: 5–146, 5–147.2, 5–147.3).

In order to ensure comparability of the study populations we analyzed sociodemographic structures as well as their respective medical history within 12 months prior to the cataract extraction. We considered the most relevant comorbidities as well as relevant drugs with a potential impact on PCO development/postoperative complications. Relevant comorbidities include retinal detachment and tear (H33), glaucoma (H40), diabetes mellitus (E10–E14) and hypertension (I10–I15; Pham et al. 2004). Certain medical treatments, systemic as well as topical, can have an influence on the proliferation of cells (Guo and DiPietro 2010; Noon et al. 2013), i.e., pharmacological interventions during or after surgery with the goal of depleting or inhibiting regeneration of remaining lens epithelial cells. Systemic drugs with a potential impact on complications such as cytostatic drugs, immunosuppressant drugs like corticosteroids and selective serotonin reuptake inhibitors (SSRI), a group of drugs which is mainly prescribed in case of depression, were identified by assessing the prescriptions based on ATC codes for pharmaceutical treatment, i.e., cytostatic drugs (ATC L01 and PZN 9999092, 2566881), Cortisone (ATC: H02, S01BA, S01BB, S01BX, S01CA, S01CB) and SSRI (ATC: N06AB02–10). To control for the severity of a patient’s comorbidities, we considered the Charlson comorbidity index (Charlson et al. 1987), which predicts the 1-year mortality.

We used Fisher’s exact test and Welch’s t-test to identify significant group differences and Bonferroni correction to avoid a false rejection of the null-hypothesis due to multiple testing in the same sample.^2^ To assess the impact of the risk factors on the dependent variable PCO incidence we used a weighted multivariate logistic regression. We included age at cataract surgery, gender, the implanted IOL type and comorbidities as well as prescribed medication. Patients with a contralateral IOL implantation during the follow-up period got a higher weight in the regression analysis to counterbalance the higher risk of PCO caused by the second IOL-implantation. All analyses were performed in SAS version 9.2 using a proc. logistic model with effect coding. Statistical significance was set at *p* < 0.05.

### Costs of complications

The economic analysis compared direct costs over a period of 48 months after cataract surgery from an SHI payer perspective. Follow-up capsulotomy after IOL implantation is usually performed in outpatient settings in Germany. To calculate costs, we thus valued these interventions by means of the EBM of 2014. The number of follow-up Nd:YAG laser capsulotomies was calculated by summing up the respective invoiced EBM codes 31341^3^ per patient in each group when the relevant procedures were coded. We additionally identified all billing positions that are related to PCO treatment. These include postoperative monitoring (EBM code 31501) and postoperative services either based on the surgeon’s referral (EBM code 31724) or performed by the operating surgeon him/herself (EBM code 31725). Since we used the most recent available version of the scheme, we did not discount costs in the analysis.

## Results

### Study population and subgroup comparison

A total of 3,025 patient records with acrylic IOL implants was selected from the database as described in the methods section (Table 1). The records were grouped by the type of IOL material. From the total population, 2,078 patients obtained a hydrophobic IOL (study population A: hydrophobic IOL) and 947 patients got a hydrophilic implant (study population B: hydrophilic IOL).

**Table 1:** Selection of insured persons

Selection of insured persons		
1st selection	Continuously insured persons in SHI from 2009 to 2014: 3,248,423	
2nd selection	Continuously registered insurants in Bavaria in 2009–2014: 452,251	
3rd selection	Insurants with a cataract diagnosis (H25, H26, H28.8) in 2009–2014: 79,422	
4th selection	Insurants with a cataract surgery in 2010 and at least 18 years old at the date of surgery: 3,713	
5th selection	Insurants with an IOL implant on the same day as cataract surgery: 3,404	
6th selection	Insurants without any IOL implantation in 2009: 3,060	
7th selection	Insurants with only one type of IOL implant in 2010: 3,025	
Subgroups	Study population A hydrophobic IOL 2,078	Study population B hydrophilic IOL 947

The mean age of the study population A is slightly higher compared to group B and both populations were predominately females (A: 59.34%; B: 57.34%; Table 2). Age and gender distributions of the subgroups are shown in Table 3. In this cohort, cataract diagnosis was more prevalent in older patients. The highest rates were found in the 66–85-year-old age groups.

**Table 2:** Socio-demographic variables of the study populations

	Study population A hydrophobic IOL	Study population B hydrophilic IOL	*p* value^a^
Female	59.34%	57.34%	0.3009
Mean age (SD)	72.79 (8.41)	73.64 (8.44)	0.0099

^a^Gender: Fisher’s exact test; age: Welch’s test; local significance level* α* = 0.005 after Bonferroni correction

**Table 3:** Age and gender distributions

Age group	Study population A hydrophobic IOL				Study population B hydrophilic IOL			
	Male	Female	Total	Percentage	Male	Female	Total	Percentage
18–50	17	18	35	1.68%	12	11	23	2.43%
51–55	19	24	43	2.07%	8	5	13	1.37%
56–60	40	50	90	4.33%	26	8	34	3.59%
61–65	79	90	169	8.13%	31	26	57	6.02%
66–70	151	217	368	17.71%	60	79	139	14.68%
71–75	245	318	563	27.09%	108	163	271	28.62%
76–80	175	267	442	21.27%	95	131	226	23.86%
81–85	92	194	286	13.76%	50	86	136	14.36%
86–90	27	49	76	3.66%	13	30	43	4.54%
91–120	0	6	6	0.29%	1	4	5	0.53%
Total	845	1,233	2,078	100.00%	404	543	947	100.00%

The most relevant comorbidities for an IOL implantation and the Charlson comorbidity index were analyzed for a period of 12 months before the surgery (Table 4). A large proportion of patients suffered from hypertension (>70%) or diabetes (~30%). Glaucoma was diagnosed in around 18% of all cases in each group, whereas a retinal detachment and a retinal tear appeared less frequently (< 2%). The prevalence rates for diabetes and hypertension were as expected, given the high mean age of both populations. It is known, that older patients suffering from diabetes may have an increased risk of postoperative complications and decreased postoperative visual functions (Lara-Smalling and Cakiner-Egilmez 2014) as well as for the development of PCO in the short-term (Hayashi et al. 2002; Ebihara et al. 2006; Raj et al. 2007); nevertheless, recent studies (Nekolová et al. 2008; Praveen et al. 2014) show, that diabetes mellitus does not increase the incidence of PCO in the long term, i.e. after 4, respectively 7, years. As this study is based on ICD diagnosis coding, there is no information available on severity of diabetes or diabetic symptoms, but also Hayashi et al. (2002) and Praveen et al. (2014) found that the diabetic severity has no influence on the development of PCO. Although the percentage of diabetic patients is higher in population B, diabetes is seen as a confounder without relation to PCO incidence. The mean score of the Charlson comorbidity index is 2.00 for study population A and 2.03 for study population B.

**Table 4:** Morbidity structure of the study populations

Comorbidity	Study population A hydrophobic IOL		Study population B hydrophilic IOL		*p* value^a^
	Total	Percentage	Total	Percentage	
Retinal detachment and tear	41	1.97%	17	1.80%	0.8864
Glaucoma	376	18.09%	183	19.32%	0.4194
Diabetes mellitus	615	29.60%	305	32.21%	0.1477
Hypertension	1,536	73.92%	701	74.02%	0.9644
Charlson index, mean (SD)	2.00 (1.96)		2.03 (1.99)		0.6947

^a^Comorbidity: Fisher’s exact test; Charlson index: Welch’s test; local significance level* α* = 0.005 after Bonferroni correction

Table 5 depicts the rates of patients with prescriptions of drugs that potentially influence PCO incidence and surgery outcomes. About half of the patients received cortisone (topical medication) in the year before the surgery. About 4–5% of patients also received SSRI, whereas cytostatic drugs rarely appear. Using Fisher’s exact test and Bonferroni correction to adjust for multiple testing, we found no between-group differences in the preoperative characteristics at a 5% global significance level, so comparability of the populations could be assumed.

**Table 5:** Medication of the study populations

Medication	Study population A hydrophobic IOL		Study population B hydrophilic IOL		*p* value^a^
	Total	Percentage	Total	Percentage	
Cytostatic	21	1.01%	6	0.63%	0.4054
SSRI	109	5.25%	38	4.01%	0.1711
Cortisone	1129	54.33%	471	49.74%	0.0205

^a^Fisher’s exact test; local significance level* α* = 0.005 after Bonferroni correction

### Complications after cataract surgery

Since PCO is the most frequent complication after IOL implantation, its incidence rate is a valid indicator of treatment quality. As shown in Table 6, PCO is treated in more than 99% of all cases by Nd:YAG laser capsulotomy; thus, in line with the literature (Findl et al. 2007), PCO treatment can be sufficiently referred to as Nd:YAG laser capsulotomy.

**Table 6:** Types of PCO treatment

	Study population A hydrophobic IOL		Study population B hydrophilic IOL	
	Total	Percentage	Total	Percentage
PCO treatment overall	656		536	
Nd:YAG laser capsulotomy	651	99.24%	532	99.25%
Surgical intervention	5	0.76%	4	0.75%

**Fig. 1:**
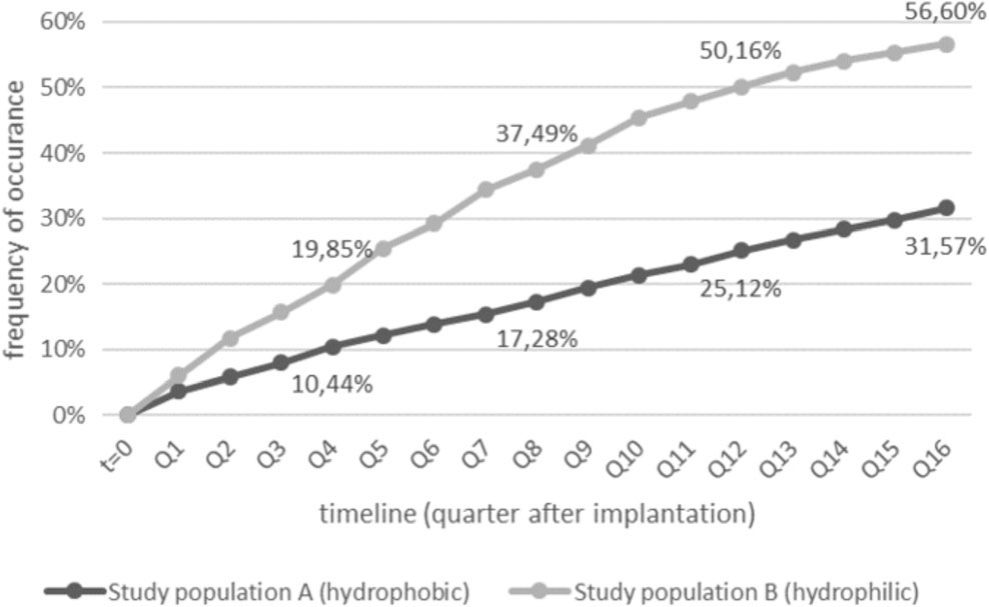
Patients with treatment of PCO (Nd:YAG laser capsulotomy or surgical intervention)

Within 4 years after IOL implantation, the frequency of PCO treatment was significantly lower in the group of patients with a hydrophobic IOL implant (*n*_abs_ = 656; 31.57%) compared with patients in study population B (*n*_abs_ = 536; 56.60%; *p* < 0.0001). Figure 1 presents the proportions of the patients who underwent PCO treatment by Nd:YAG laser capsulotomy or surgical intervention within 4 years after the implantation of the IOL. The curves confirm that PCO treatment occurred more frequently in the hydrophilic IOL group in every quarter after cataract surgery.

To exclude potential confounders, other adverse effects that required follow-up surgery are taken into account as control variables as shown in Table 7. There was no statistically significant difference between the groups, so that PCO could be considered as the single indicator for the treatment quality outcome.

**Table 7:** Postoperative complications (control variables)

	Study population A hydrophobic IOL		Study population B hydrophilic IOL		*p* value^a^
	Total	Percentage	Total	Percentage	
Glaucoma	71	3.42%	24	2.53%	0.2172
Vitreoretinal intervention	56	2.69%	20	2.11%	0.3821
Explantation and secondary implantation of an IOL	57	2.74%	24	2.53%	0.8087

^a^Fisher’s exact test

The regression results and odds ratios in Table 8 show that the type of IOL implant exerts the strongest negative impact on expected PCO incidence rates (−0.5143, *p* < 0.0001). For patients with hydrophobic IOL implants, the likelihood is reduced by a factor of 0.3575. Sex, i.e. male (−0.1775, *p* < 0.0001, OR 0.7012) and a diagnosed hypertension (−0.0915, *p* < 0.05, OR 0.8328) are moderately related to a lower PCO development. Glaucoma (0.0833, *p* < 0.05) as well as a prescription of cytostatic drugs (0.3972, *p* < 0.05) are related to an increased likelihood of PCO. Age, diabetes, retinal detachment and tear, as well as the prescription of cortisone and SSRI are not significantly related to PCO incidence.

**Table 8:** Results of the regression analysis (weighted logistic regression)

Variables	Coefficient	Odds ratio
Lens type	−0.5143***	0.3575
Socio-demographics		
Age	−0.0031	0.9969
Gender (male)	−0.1775***	0.7012
Comorbidities		
Glaucoma	0.0833*	1.1813
Diabetes	0.0016	1.0031
Hypertension	−0.0915*	0.8328
Retinal detachment and tear	0.0198	1.0404
Medication		
Cytostatic	0.3972*	2.2131
SSRI	−0.1182	0.7895
Cortisone	0.0356	1.0738

* p < 0.05; ** p < 0.001; *** p < 0.0001

### Economic impact

Due to the higher PCO incidence in hydrophilic group, Nd:YAG laser capsulotomy after cataract surgery was subsequently more often performed in this group as well. In fact, 0.51 fee schedule positions per patient were billed for the hydrophobic group and 0.9 per patient for the hydrophilic group (Table 9). Associated treatments are likewise more frequent in the hydrophilic study population B. 0.46 positions per patient accounted for postsurgical monitoring in the hydrophobic, compared to 0.82 positions in the hydrophilic group. Furthermore, a considerably higher sum of billed positions per patient was scheduled for postoperative treatments conducted by the surgeon in the hydrophilic group (0.37 vs. 0.66). The average costs per patient (i.e. not per treatment) were 50.03 € for the hydrophobic group and 87.81 € for the hydrophilic group.

**Table 9:** Mean costs of PCO treatment by Nd:YAG laser capsulotomy

	Type of treatment	Study population A hydrophobic IOL		Study population B hydrophilic IOL	
		Sum	Per patient	Sum	Per patient
Total number of patients		2078		947	
Number of billed fee schedule positions	Laser-surgical intervention of category W1	1058	0.51	849	0.90
	Postsurgical monitoring	961	0.46	773	0.82
	Postoperative treatment (referral)	83	0.04	41	0.04
	Postoperative treatment (surgeon)	773	0.37	628	0.66
Cumulative costs per treatment	Laser-surgical intervention of category W1 (78.68 € per fee schedule position)^a^	83,243.44 €	40.06 €	66,799.32 €	70.54 €
	Postsurgical monitoring (14.69 € per fee schedule position)^b^	14,117.09 €	6.79 €	11,355.37 €	11.99 €
	Postoperative treatment (referral) (13.46 € per fee schedule position)^c^	1117.18 €	0.54 €	551.86 €	0.58 €
	Postoperative treatment (surgeon) (7.09 € per fee schedule position)^d^	5480.57 €	2.64 €	4452.52 €	4.70 €
Costs PCO treatment		103,958.28 €	50.03 €	83,159.07 €	87.81 €

^a^EBM 31341

^b^BM 31501

^c^EBM 31724

^d^EBM 31725

The average postoperative costs due to PCO treatment by Nd:YAG laser capsulotomy for patients with hydrophilic IOL are about 75% higher compared to patients with hydrophobic IOL implants. Within the scope of this cost analysis the base flat rate for ophthalmologists^4^ is not considered because it is not clearly attributable to the PCO treatment. Assuming that these contacts are also related to PCO treatment, the economical advantageousness of the hydrophobic lens would increase even more.

Occasionally, there is a need for another IOL implantation in the contralateral eye. Such an additional intervention has to be considered in the economic impact assessment. Since the ratio of cataract patients that underwent a contralateral IOL implantation in the follow-up period differs between the groups (Table 10), the associated PCO risk and the necessity of its treatment differ as well.

**Table 10:** Contralateral IOL-implantation in the follow-up period

Contralateral IOL-implantation	Study population A hydrophobic IOL	Study population B hydrophilic IOL	*p* value^a^
In 1-year follow-up	56.59%	50.69%	0.0028
In 2-year follow-up	60.30%	55.23%	0.0096
In 3-year follow-up	61.93%	58.82%	0.1080
In 4-year follow-up	64.29%	61.35%	0.1222

^a^Fisher’s exact test

If the follow-up time of the contralateral IOL implantation is measured too, this results in an average 1.57-year follow-up of the contralateral eye in population A and 1.53-year in population B (*p* = 0.1222). Considering both eyes, this yields a summarized average follow-up time of 5.57 years (population A) and 5.53 years (population B), respectively. Since a contralateral IOL-implantation increases the risk for a PCO treatment, the average costs for patients with hydrophobic IOL implants are overestimated by around 2.6%^5^ and therefore should be decreased by this factor compared to hydrophilic IOL.^6^ This would result in average costs of 48.72 € for population A.

## Discussion

This retrospective analysis was performed to compare the incidence of PCO measured by Nd:YAG laser capsulotomy or surgical intervention after implantation of either hydrophobic or hydrophilic IOLs following cataract extraction. Additionally, other postoperative adverse events such as glaucoma, vitreoretinal interventions and the explantation and replacement of the IOL were considered as control variables. The focus of the economic analysis was to assess the follow-up costs related to the different implanted IOL materials and the potentially following complications from an SHI perspective.

Most of the available evidence to compare IOL-types is provided by retrospective cohort studies or randomized controlled trials (RCT) with small sample sizes under artificial controlled study conditions mainly with short follow-up times. Under these conditions, hydrophobic acrylic lens material (compared to hydrophilic IOLs) was found to be related to a lower risk of PCO development measured by the rate of Nd:YAG laser capsulotomy compared to hydrophilic acrylic IOL material (34% vs. 49%, *p* = 0.04; Schriefl et al. 2015).

The meta-analysis of Li et al. (2013) finds a relative risk of 6.96 for Nd:YAG laser capsulotomy 2 years after cataract surgery, when hydrophilic IOLs are compared with hydrophobic IOLs. Several retrospective studies come to similar conclusions. Gauthier, Lafuma, Laurendeau and Berdeaux (2010) found that the rate of Nd:YAG laser capsulotomy after bilateral hydrophobic IOL implantation was 8.8%, whereas it reached 37.5% after bilateral hydrophilic IOL implantation in a 2-year follow-up of 312 eyes. Boureau et al. (2009b) found, that 45.4% of patients with hydrophilic implants had undergone Nd:YAG laser capsulotomy, while the amount was significantly lower (*p* < 0.001) if a hydrophobic IOL was implanted (13%/23.4%). However, the summarized cohort or RCT to compare rates of Nd:YAG laser capsulotomy in relation to different IOL had small sample sizes (675 patients on the average) and follow-up periods of 1–3 years after the first IOL implantation (Auffarth et al. 2004; Boureau et al. 2009b; Gauthier et al. 2010; Vasavada et al. 2011; Li et al. 2013). Many scientific conclusions were made accordingly (e.g. as Auffarth et al. (2004) that state, incidence rates of Nd:YAG laser capsulotomy do not considerably decrease over a 3-year follow up period).

In contrast, the present study assessed a population of 3,025 patients in a 4-year-follow-up under real world conditions. Accordingly, our results add to the scientific knowledge of Nd:YAG laser capsulotomies within longer time frames and with a large study population under real world conditions.

A considerable part of cataract-associated costs results from complications of the surgery and its treatments as well as related secondary adverse events; however, economic analysis from a SHI perspective has been very limited up to now. Based on a retrospective, multicenter study including 767 eyes treated, Boureau et al. (2009a, b) calculated a model of patients’ lifetime costs from a French SHI perspective for populations that either had hydrophobic or hydrophilic acrylic IOL implanted. Direct and indirect cost estimates were derived from official French documents, published literature and expert declarations. The authors found significantly higher adjusted risk ratios of undergoing an Nd:YAG laser capsulotomy (RR: 5.1; *p* < 0.0001) for hydrophilic lenses compared to the hydrophobic IOL. For a linear extrapolation of Nd:YAG laser capsulotomy rates from 5 to 26 years of follow-up, total costs of treatment and management of complications per patient^7^ were more than 3-times higher for patients with a hydrophilic lens implant (268.90 €) compared to the best performing hydrophobic implant (84.78 €) in the study. Indirect costs, associated to the risk of blindness, account for a considerable amount of about 42% of total costs. In the budget analyses, savings of 21.936.621 € from a French SHI perspective were estimated if all patients undergoing cataract extraction in 2005 switched to the better-performing IOL material (Boureau et al. 2009a). Due to the differences in national economic regulation, a generalizability of the results is however limited; moreover, the modeling approach is susceptible to uncertainties with regard to variable quality of the information considered. Complication rates have not been collected from medical records or claims data but estimated based on the literature, which might cause some imprecision. Smith et al. (2005) compared cost-effectiveness ratios, i.e. costs per patient successfully treated without Nd:YAG laser capsulotomy, of four types of IOL material in a retrospective cross-country study. Their results show that hydrophobic IOLs seem to be more cost-effective than other materials in most countries. Costs were estimated on the basis on official documents and the mean total costs per successfully treated patient and IOL-type were computed and compared. However, the differences in regulatory frameworks and reimbursements between countries influence the economic estimates and limit the comparability of the results.

Comparing the follow-up costs associated with PCO treatment by Nd:YAG laser capsulotomy and post-operative monitoring and services, our results point in the same direction as previous studies (Smith et al. 2005; Boureau et al. 2009a). The difference in average costs per patient indicates the superiority of hydrophobic lens material from an economic point of view.

Our results add to the scarce health economic evidence related to treatment of complications after cataract surgery regarding two main aspects. Firstly, in contrast to available research (Smith et al. 2005; Boureau et al. 2009a) our health economic analysis is based on longitudinal claims data. To our knowledge, there is no study that compares different IOL material types combined with a cost analysis based on real world claims data. Results from real world economic analysis is particularly important for payers (i.e. statutory health insurance in Germany) to make value-basedcoverage and re-imbursementdecisions (Garrison et al. 2007). The database we used is characterized by a good accordance with the German population regarding sociodemographic indicators, morbidity, mortality and medication use. Thus, generalizability of the results is superior compared to studies including records from selected health care providers. Persistence with the database is high (78.5% of the insurants can be observed from 2009 to 2013); hence, longitudinal analysis is very reliable (Andersohn and Walker 2016). In contrast to retrospective multicenter post-test studies that account for most of the outlined evidence of IOL-related outcomes research, SHI claims data reflect daily practice and cover the whole spectrum of treatments and prescriptions by different health care providers (Pigeot et al. 2008). It guarantees 100% study participation proportions, which reduces potential selection biases; thus, it is possible to derive reliable actual SHI cost estimates.

This study has some limitations that are related to the structure and characteristics of claims data. Claims data are collected for billing purposes and occasionally there are inconsistencies and implausible values (Pigeot et al. 2008). The choice of potential confounders is limited to the available variables and a prospective design is not possible (Pigeot et al. 2008). Moreover, problems regarding the validity of diagnoses and procedure codes as well as coding errors are well-known problems in terms of billing data (Swart 2014). A differentiated coding for hydrophobic and hydrophilic acrylic IOL implantation is to our knowledge limited in Germany to the federal state of Bavaria, which may limit the generalizability of our results. However, the German SHI benefits for cataract surgery, pre-and post-operative care including treatment of complications are comparable to other federal states and related to the national reimbursement catalog for public medical services (EBM catalog). In addition, no restrictions exist regarding which IOL material should be implanted. In all German federal states, cataract surgeons can select the IOL material they prefer and coverage is granted by public reimbursement; therefore, there is no reason to assume that treatment and associated effects vary considerably in other federal states.

## Conclusion

Cataract extraction as one of the most common surgical interventions in the developed world is an established and safe surgical procedure. The benefit of IOL implantation has been proven for decades; nevertheless, considering the high prevalence of cataract, the economic burden associated with treatment of long-term postoperative complications after cataract surgery is of great relevance for health budgets, also for the German SHI. This study identified different long-term outcomes under real world conditions highly related to the IOL material used. Hydrophobic acrylic lenses seem to be superior with regard to both medical and economic results compared to hydrophilic acrylic IOLs. Considering the necessity of follow-up treatments and intangible costs, the hydrophobic lens implant is likely to be preferable also from the patient’s perspective. More research on patient-reported outcomes is necessary to confirm this hypothesis.

## Footnotes

1 Up to 9 years after surgery

2 The Bonferroni correction is based on the idea that local significance levels for each of the independent hypothesis tests can be divided by the total number of hypotheses in order to test a global significance level.

3 laser-surgical intervention of category W1

4 EBM 06210, 06211, 06212 (12.87 € to 15.60 €)

5 Due to the 2.6% longer follow-up time of the contralateral IOL implantation for population A (1.57 year vs. 1.53 year)

6 Assuming the costs increase in an equal proportion

7 5% discount rate
